# What information and the extent of information to be provided in an informed assent/consent form of pediatric drug trials

**DOI:** 10.1186/s12910-022-00856-y

**Published:** 2022-11-16

**Authors:** Nut Koonrungsesomboon, Pimlak Charoenkwan, Rungrote Natesirinilkul, Kanda Fanhchaksai, Wannachai Sakuludomkan, Nimit Morakote

**Affiliations:** 1 Department of Pharmacology, Faculty of Medicine, Chiang Mai University, Chiang Mai, 50200 Thailand; 2 Clinical Research Center for Food and Herbal Product Trials and Development (CR-FAH), Faculty of Medicine, Chiang Mai University, Chiang Mai, Thailand; 3 Musculoskeletal Science and Translational Research (MSTR) Center, Faculty of Medicine, Chiang Mai University, Chiang Mai, Thailand; 4 Department of Pediatrics, Faculty of Medicine, Chiang Mai University, Chiang Mai, Thailand; 5 Thalassemia and Hematology Center, Faculty of Medicine, Chiang Mai University, Chiang Mai, Thailand; 6 Department of Parasitology, Faculty of Medicine, Chiang Mai University, Chiang Mai, Thailand

**Keywords:** Informed consent, Parental consent, Assent, Consent forms, Ethics, Clinical trials

## Abstract

**Background:**

This study aimed to determine the elements and the extent of information that child participants and their parents would like to read in an informed assent form (IAF)/informed consent form (ICF) of a pediatric drug trial.

**Methods:**

A descriptive survey was conducted to determine the perceived importance of each element of the ICF content from child participants and their parents who underwent informed assent/consent of a multi-center pediatric drug trial. The respondents were asked to indicate the level of importance of each item in a questionnaire, by giving a rating scale from 1 (not important) to 5 (very important).

**Results:**

A total of 22 families, 17 child participants with the diagnosis of hematology or oncology diseases and 27 parents, were enrolled. Among 30 items, risk–benefit aspects (i.e., direct health benefit [mean: 4.71 for child respondents, 4.89 for parent respondents], indirect/societal benefit [mean: 4.65, 4.85], major foreseeable risk [mean: 4.47, 4.78], post-trial benefit/provision [mean: 4.59, 4.74], and all adverse effects of the drug including uncommon adverse effects [mean: 4.53, 4.74]) were perceived to be of most concerning items from both child participants’ and parents’ viewpoint. None of the items were considered ‘slightly important’ or lower by more than 20% of the respondents.

**Conclusions:**

For pediatric drug trials, risk–benefit information (including direct health benefit, indirect/societal benefit, and post-trial benefit/provision, as well as major foreseeable risk and adverse effects of the drug) should be made a salient feature of an IAF/ICF. This empirical data could help related stakeholders arrange essential information in order of importance and tailor an IAF/ICF to better suit child participants’ and parents’ needs, particularly for pediatric drug trials involving children with the diagnosis of hematology or oncology diseases.

## Key points

- Informed assent/consent remains one of the most challenging issues in pediatric drug trials, with no trustworthy clue or guidance to determine what type of information and the extent of information to be provided in an informed assent/consent form.
- Based on our empirical data, risk–benefit aspects related to trial participation were perceived to be more important than general information or technical details of the trial, although all the elements are deemed useful, to a certain degree, to both child participants and their parents.
- Both child participants and their parents did not want to read an unduly long form, with the maximum acceptable page length of no more than 5–8 pages.

## Background

In pediatric clinical trials, enrollment of child participants generally requires parental consent and child assent [1, 2]. Sufficient information in an informed consent form (ICF) is of vital importance to enable the parents of potential child participants to make valid informed decisions, for the best interest of their child, whether or not to let him/her participate in a trial [3, 4]. An ICF for parental consent is typically almost identical to an ICF used for adult participants, except that it refers to the child as a research participant and asks for parental consent as a proxy decision-maker. What information to be provided in an ICF generally refers to the essential elements of the ICF content described in the major ethical guidelines and regulations (i.e., ICH E6(R2), Declaration of Helsinki 2013, and 45 CFR 46.116). However, the extent of information to be considered ‘sufficient’ has remained the subject of debate [5–7]. Disclosure of every single detail of information about the trial may unnecessarily complicate and lengthen an ICF, so that the parents may be overwhelmed by extraneous information while crucial information is often out of sight [8, 9].

For child assent, such issues are even more challenging, with no explicit guidelines clearly outlining the scope of what would be considered necessary information to be provided in an informed assent form (IAF) [10–12]. The responsibility for defining what constitutes adequate information in an IAF generally resides with the institutional review board (IRB)/independent ethics committee (IEC), resulting in disparities in IAF requirements across different IRBs/IECs [13]. What information and the extent of information that potential child participants want to know are often construed in different ways [14–16]. As such, informed assent remains one of the most challenging issues in pediatric clinical trials, with no trustworthy clue or guidance to determine what type of information and the extent of information to be provided in an IAF [17, 18].

Little empirical research is available to provide proper guidance on what information should be provided to child participants as well as the extent of information child participants and their parents would like to know [19, 20]. There may be a discrepancy between what they actually want to know and what the IAF/ICF does provide [21]. Recently, the Forum for Ethical Review Committees in the Asia and Western Pacific region (FERCAP) has published the results of a multi-country survey of ‘what information and the extent of information research participants need in informed consent forms’ across seven FERCAP-member countries, including Thailand [22]. However, the FERCAP survey did not include those involving pediatric research; child and parental perceptions towards information provided in an IAF/ICF have not yet been explored. It would be of value to determine the information needs among child participants and their parents who are invited to take part in pediatric research, particularly in high-risk research like clinical trials of investigational new drugs.

The objective of the present study was to determine the elements and the extent of information that child participants with the diagnosis of hematology or oncology diseases and their parents would like to read in an IAF/ICF of a pediatric drug trial. It was expected to provide empirical data that can help investigators and sponsors tailor IAFs/ICFs toward this specific group of population’s needs and perspectives.

## Methods

### Study design and eligible criteria

This single-center, cross-sectional, descriptive survey was conducted at the Faculty of Medicine, Chiang Mai University, between April and July 2021. Children (aged > 7 years) with the diagnosis of hematology or oncology diseases (defined according to ICD10: D50-D77 and C00-D48) and their parents (aged > 20 years) who underwent informed assent/consent of a multi-center pediatric drug trial were enrolled. Individuals (either child participants or their parents) who refused to answer the questionnaire for any reason or had communication difficulties due to language problems (including unable to read the Thai language) or cognitive disabilities, as judged by a person obtaining informed assent/consent, were excluded.

### Questionnaire

The original questionnaire was used in a multi-country survey across seven FERCAP-member countries, including Thailand [22]. The questionnaire was modified following the Guidance and Template of Informed Consent Form for Clinical Trials in Thailand [23]; it consisted of 30 survey items relevant to pediatric drug trials. The respondents were asked to indicate the level of importance of each item (in other words, the extent of information or the depth of details of each item to be provided in an IAF/ICF), based on their perspectives, by giving a rating scale from 1 (not important) to 5 (very important) using a modified Likert scale. Open-ended questions were placed at the end of the questionnaire allowing the respondents to write down any additional information needs, or any further suggestions related to an IAF/ICF.

### Recruitment of the respondents and data collection

Eligible children and parents were contacted, during their regular follow-up care, at the outpatient clinic, Department of Pediatrics, Faculty of Medicine, Chiang Mai University, Chiang Mai, Thailand. They were instructed that: (1) they could refuse to take part in this study, (2) they could skip any items/questions that they were unwilling to answer, and (3) they would not be treated differently as a result of their given answers. If each individual agreed to take part in this survey, the subject number would be assigned, and the paper-based questionnaire was then distributed. Written assent/consent was obtained in a private room prior to the distribution of the questionnaire.

The child and parent respondents were required to reread an IAF/ICF of the multi-center pediatric drug trial that they or their child used to participate in or were participating in. The IAF/ICF document given to each respondent corresponded to his/her current age/status at the date of enrollment in this survey study. After reading the documents, they were asked to complete the questionnaire in a private setting, arranged by the investigators, by themselves, or with the assistance of research staff. The child and parent respondents could consult the research staff if they would have had any questions related to the questionnaire.

### Data analysis

Descriptive statistics were used to describe the demographic characteristics of the respondents and the perceived importance of each item in the questionnaire. The results are presented as the frequency with percentage, mean with standard deviation (SD), or median with interquartile range (IQR), as appropriate. Items would be of most concern or of less concern if the value was more than ‘mean + 1 SD’ or less than ‘mean − 1 SD’, respectively. A *p* value of less than 0.05 was considered to indicate statistical significance. All statistical analyses were done using IBM SPSS Statistics for Windows, Version 22.0. Armonk, NY: IBM Corp. Released 2013.

## Results

A total of 22 families who visited the outpatient clinic during April–July 2021 were enrolled in this study. The demographic characteristics of the respondents are shown in Table 1. There were 17 child respondents, with a mean age of 13.2 ± 5.5 years (median, 14 years; IQR, 8–18 years) and 27 parent respondents, with a mean age of 40.0 ± 9.9 years (median, 41 years; IQR, 31–48 years). More than half of the parent respondents had a high-school level of education or lower (55.6%). Most of the respondents were involved in a process of informed consent of phase 2 or 3 multi-center pediatric drug trials, with the exception of only 1 parent respondent whose child used to participate in a phase 4 clinical trial.

**Table 1:** Demographic characteristics of the respondents

	Child respondents (n = 17)	Parent respondents (n = 27)
Age (year)	13.2 ± 5.5	40.0 ± 9.9
Gender		
Male	14 (82.4%)	10 (37.0%)
Female	3 (17.6%)	17 (63.0%)
Education		
High school (or lower)		15 (55.6%)
Bachelor (or equivalent)		10 (37.0%)
Master (or higher)		2 (7.4%)
Underlying diseases/conditions of child participants		
Beta-thalassemia	7 (41.2%)	10 (37.0%)
Hereditary factor VIII deficiency	6 (35.3%)	10 (37.0%)
Hereditary factor IX deficiency	2 (11.8%)	4 (14.8%)
Hereditary hemolytic anemia	1 (5.9%)	1 (3.7%)
Embolism and thrombosis of vena cava	0 (0.0%)	2 (7.4%)
Chronic myeloid leukemia	1 (5.9%)	0 (0.0%)
Clinical phase		
Phase 2	9 (52.9%)	10 (37.0%)
Phase 2/3	1 (5.9%)	2 (7.4%)
Phase 3	7 (41.2%)	14 (51.9%)
Phase 4	0 (0.0%)	1 (3.7%)
Time from informed assent/consent of the pediatric drug trial to enrollment of this study (month)	55.2 ± 14.1	47.7 ± 17.9

**Fig. 1:**
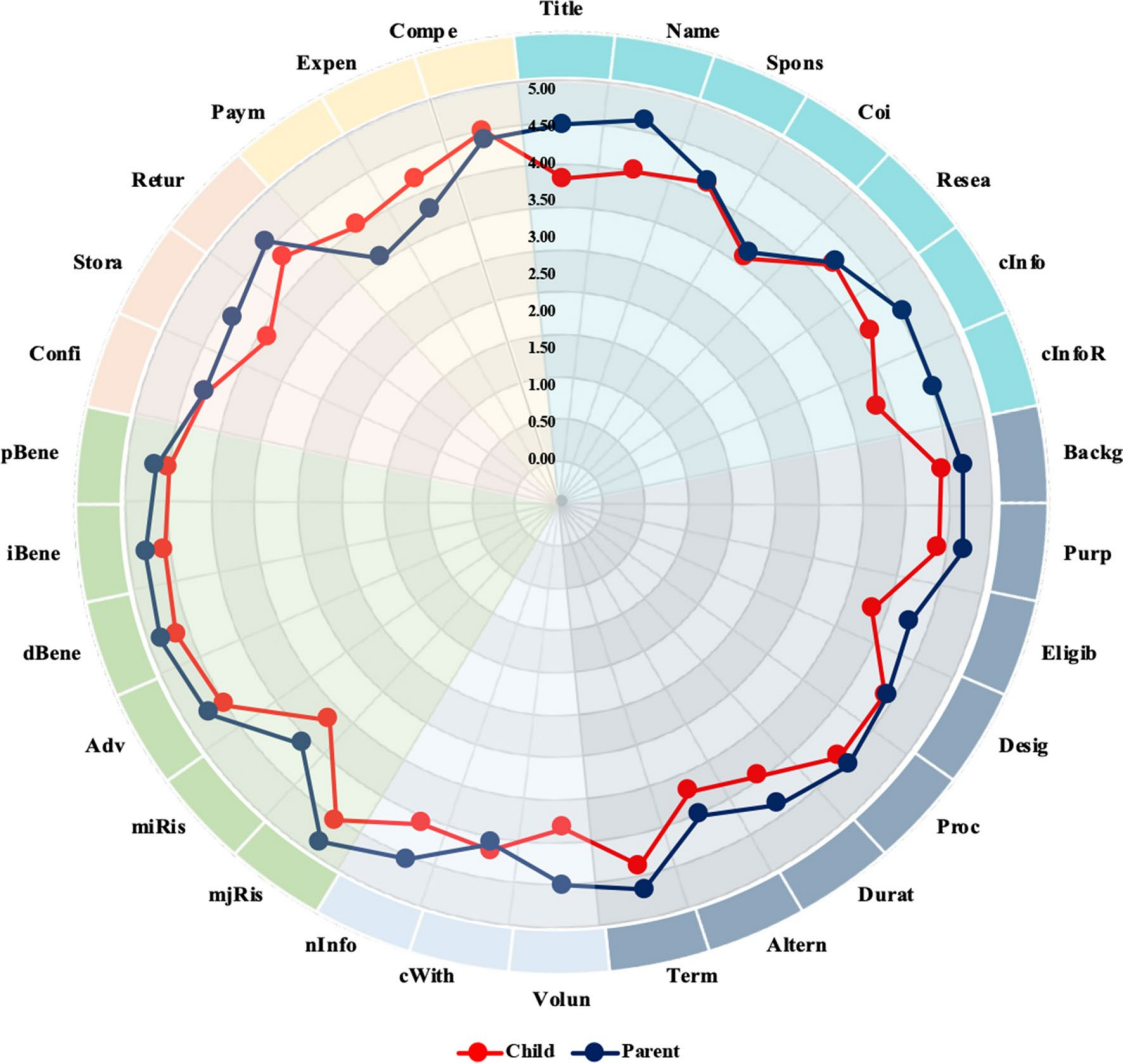
Information needs of the child and parent respondents. Each dot represents the mean score of each item

**Fig. 2:**
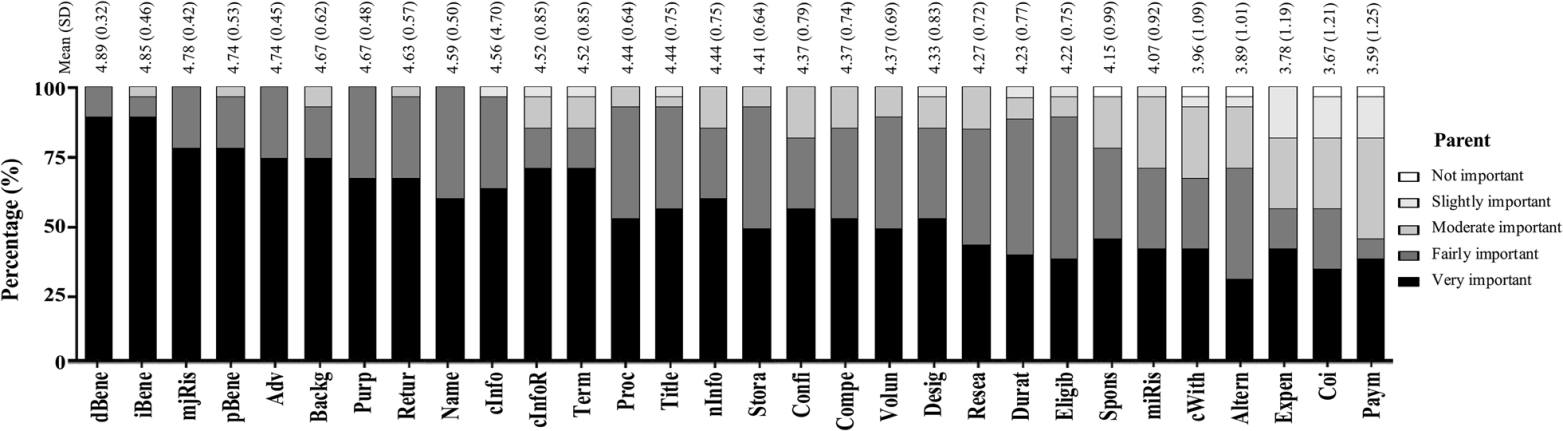
The perceived importance of each item of the parent respondents

Overall, the parent respondents wanted to know most elements of the ICF content required (Table 2; Fig. 1), with mean scores ranging from 4.89 (direct health benefit) to 3.59 (payment and/or remuneration). Five items that were rated as ‘very important’ (score = 5) or ‘fairly important’ (score = 4) by all the parent respondents included direct health benefit, major foreseeable risk, all adverse effects of the drug (including uncommon adverse effects), purpose of research, and name of researchers (and their affiliations) (Fig. 2). None of the items were considered ‘slightly important’ (score = 2) or lower by more than 20% of the parent respondents. Direct health benefit, indirect/societal benefit, major foreseeable risk, post-trial benefit/provision, and all adverse effects of the drug, including uncommon adverse effects, were considered to be of most concern among the parent respondents (with mean scores of 4.89, 4.85, 4.78, 4.74, and 4.74, respectively). In contrast, items about consequences of withdrawal, alternative courses of treatment, anticipated expense, conflict of interest, and payment and/or remuneration were considered to be of relatively less concern (with mean scores of 3.96, 3.89, 3.78, 3.67, and 3.59, respectively). The maximum page length in the ICF that the parent respondents preferred to read was 8.42 ± 4.93 pages (median, 7.5 pages; IQR, 5–10 pages).

**Table 2:** The element and extent of information that the child and parent respondents wanted to receive

Elements	Abbreviation	Extent of information							
		Child respondents (n = 17)				Parent respondents (n = 27)			
		Mean	SD	Median	IQR	Mean	SD	Median	IQR
1. General items									
1.1 Title of research	Title	3.82	1.19	4	3–5	4.44	0.75	5	4–5
1.2 Name of researchers (and their affiliation)	Name	4.00	1.17	4	3–5	4.59	0.50	5	4–5
1.3 Source of funds and sponsors	Spons	4.13	1.03	4.5	3–5	4.15	0.99	4	4–5
1.4 Conflict of interest	Coi	3.59	1.12	4	3–4.5	3.67	1.21	4	3–5
1.5 Recognition that this is research	Resea	4.24	0.75	4	4–5	4.27	0.72	4	4–5
1.6 Contact information regarding the trial	cInfo	4.12	1.05	4	3.5–5	4.56	0.70	5	4–5
1.7 Contact information about the participant’s right	cInfoR	3.82	1.13	4	3–5	4.52	0.85	5	4–5
2. Study-specific items									
2.1 Background and rationale of research as well as information about investigational new drugs	Backg	4.41	0.71	5	4–5	4.67	0.62	5	4–5
2.2 Purpose of research	Purp	4.38	0.72	4.5	4–5	4.67	0.48	5	4–5
2.3 Eligibility of the participant	Eligib	3.76	0.83	4	3–4	4.22	0.75	4	4–5
2.4 Study design of research	Desig	4.31	0.87	5	3.25–5	4.33	0.83	5	4–5
2.5 Procedure and schedule	Proc	4.29	0.92	5	4–5	4.44	0.64	5	4–5
2.6 Duration of the participant’s participation	Durat	3.82	1.13	4	3–5	4.23	0.77	4	4–5
2.7 Alternative courses of treatment	Altern	3.59	1.46	4	3–5	3.89	1.01	4	3–5
2.8 Criteria for termination	Term	4.24	1.09	5	4–5	4.52	0.85	5	4–5
3. Items related to the participant’s right									
3.1 Voluntary participation	Volun	3.71	1.26	4	3–5	4.37	0.69	4	4–5
3.2 Consequence of withdrawal	cWith	4.06	0.97	4	3–5	3.96	1.09	4	3–5
3.3 Right to receive new information	nInfo	4.00	1.12	4	3–5	4.44	0.75	5	4–5
4. Items related to risk–benefit									
4.1 Major foreseeable risk	mjRis	4.47	0.80	5	4–5	4.78	0.42	5	5–5
4.2 Minor foreseeable risk	miRis	3.65	1.06	3	3–5	4.07	0.92	4	3–5
4.3 All adverse effects, including uncommon adverse effects	Adv	4.53	0.72	5	4–5	4.74	0.45	5	4–5
4.4 Direct health benefit	dBene	4.71	0.47	5	4–5	4.89	0.32	5	5–5
4.5 Indirect/societal benefit	iBene	4.65	0.61	5	4–5	4.85	0.46	5	5–5
4.6 Post-trial benefit/provision	pBene	4.59	0.62	5	4–5	4.74	0.53	5	5–5
5. Items related to data and sample storage									
5.1 Confidentiality and the limit of confidentiality	Confi	4.35	0.86	5	3–5	4.37	0.79	5	4–5
5.2 Storage of biospecimens	Stora	3.94	0.83	4	3–5	4.41	0.64	4	4–5
5.3 Return of research results	Retur	4.35	0.79	4	4–5	4.63	0.57	5	4–5
6. Items related to monetary issues									
6.1 Payment and/or remuneration	Paym	4.06	0.97	4	3–5	3.59	1.25	3	3–5
6.2 Anticipated expense	Expen	4.19	0.98	4.5	3.25–5	3.78	1.19	4	3–5
6.3 Compensation for injury	Compe	4.47	0.94	5	4–5	4.37	0.74	5	4–5

IQR, interquartile range; SD, standard deviation

**Fig. 3:**
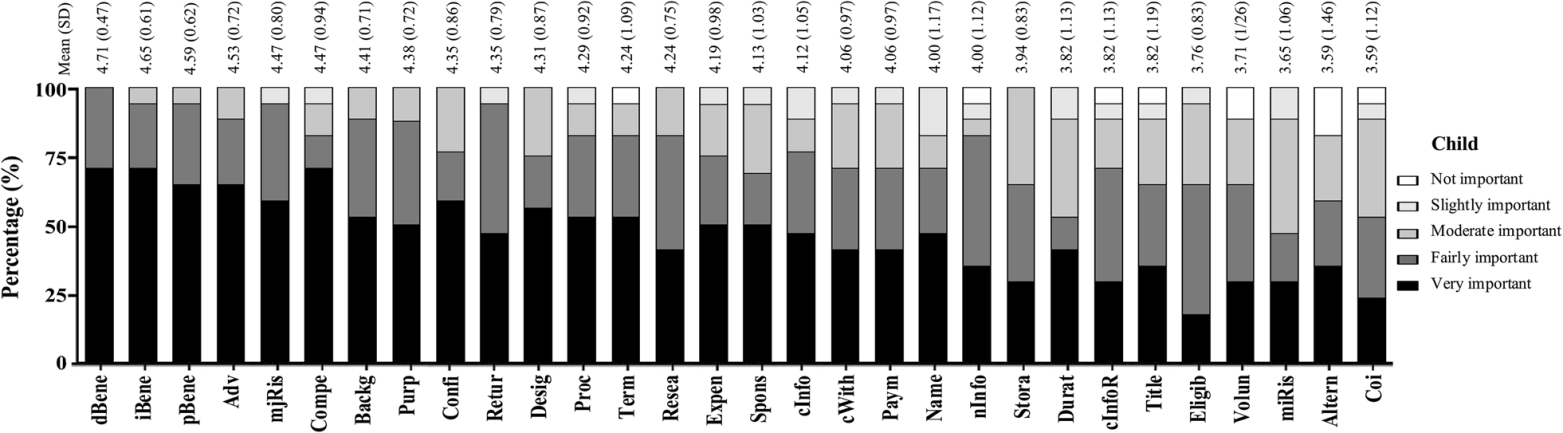
The perceived importance of each item of the child respondents

Among the child respondents, most elements of the ICF content were also considered to be of importance based on their perception (Table 2; Fig. 1), with mean scores ranging from 4.71 (direct health benefit) to 3.59 (conflict of interest). Direct health benefit was the only item that was rated as ‘very important’ or ‘fairly important’ by all the child respondents (Fig. 3). None of the items were considered ‘slightly important’ or lower by more than 20% of the child respondents. Direct health benefit, indirect/societal benefit, post-trial benefit/provision, and all adverse effects of the drug, including uncommon adverse effects, were considered to be of most concern among the child respondents (with mean scores of 4.71, 4.65, 4.59, and 4.53, respectively). In contrast, items about the storage of biospecimens, duration of the participant’s participation, contact information about the participant’s right, the title of research, eligibility of the participant, voluntary participation, minor foreseeable risk, alternative courses of treatment, and conflict of interest were considered to be of relatively less concern (with mean scores of 3.94, 3.82, 3.82, 3.82, 3.76, 3.71, 3.65, 3.59, and 3.59, respectively). The maximum, acceptable page length in the IAF was 5.87 ± 3.98 pages (median, 5 pages; IQR, 3–10 pages).

There were 7 relevant comments from 3 parent respondents and 1 child respondent, suggesting additional information needs. Overall, they wanted to receive more detailed information about drug intervention (n = 2) as well as its adverse effects on certain aspects (n = 2). They also had concerns about post-trial treatment/care (n = 2). One child respondent suggested that it would have been helpful to him/her if the IAF had contained summarized information about foods/drugs to be avoided during trial participation and how to manage certain common health problems (such as having a fever) while participating in a trial. In addition, there were other 3 comments (from 2 parent respondents and 1 child respondent) related to the IAF/ICF format: they mentioned that the IAF/ICF used in the trial was too technical and lengthy, and they rather wanted to read a concise and easy-to-read IAF/ICF in plain language.

## Discussion

This is the first empirical study that was specifically designed to explore the perceived importance of the ICF content and insights into an IAF/ICF among child participants and their parents. One of the major strengths of this study is that it reflects the desire and perspectives towards the IAF/ICF content/format of actual child participants and their parents, who took part in multi-center pediatric drug trials. In addition, our study was designed to let all the respondents go through an IAF/ICF(s) of the trial again before they answered the questionnaire. Hence, their responses would better represent the child participants’ and the parents’ perspectives with regard to the IAF/ICF content/format than the mock population who had never been experienced in a real informed assent/consent process of multi-center pediatric drug trials but are often enrolled, perhaps for ease of recruitment, in several previous empirical studies [24, 25]. Overall, both child and parent respondents in the present study considered most elements of the ICF content required to be necessary for their decision-making for trial participation, yet some items were perceived to be more important than others.

In the present study, both child and parent respondents regarded the risk–benefit aspects associated with trial participation to be more important than general information or technical details of pediatric drug trials. Direct health benefit, indirect/societal benefit, and post-trial benefit/provision, as well as major foreseeable risk and all adverse effects of the drug, including uncommon adverse effects, were the top five priority items of the IAF/ICF content that the respondents (both child participants and their parents) wanted to know the most. Our finding is consistent with a number of studies indicating that the risks and benefits related to trial participation are frequently perceived to be of most concern from the research participants’ points of view [22, 26, 27]. As such, in pediatric drug trials, information on risks (including infrequent adverse effects) and benefits (including post-trial benefit/provision) should be made a salient feature of an IAF/ICF and described comprehensively. Investigators may opt to disclose all possible adverse effects of the investigational new drug in a summary table, with highlights or bold fonts being applied to point out the common or serious ones [28]. Some groups of IRB/IEC experts, pediatric researchers, and child/parent advocates suggest that risk–benefit messages should be delivered in a manner that reflects how potential child participants might experience the risks and receive the benefits (e.g., will some procedures hurt them? and how will the drug help them with their suffering condition?) rather than the risks and benefits per se [12].

It can be assumed from our empirical data that an unduly long IAF/ICF of some pediatric drug trials might not be fully read either by child participants or their parents [29]. The analysis of the respondents’ acceptable page length suggests that an IAF/ICF should preferably be no more than 5–8 pages in length, and it should not exceed the 10-page length limitation. Our finding corroborates a recent change in the ethical guidelines and regulations that encourage the use of concise forms [30, 31]. A number of strategies have been proposed to enhance the concision of an IAF/ICF and make the form more comprehensible [32–37]. It is evident that a concise information sheet can be as valid as a detailed one with respect to regulatory compliance [38, 39]. Investigators may also opt to apply a participant-oriented approach that considers the importance of each item and puts emphasis on items perceived as more important than the others. Boilerplate language and unnecessarily lengthy details that may mask or dilute the main content and further obfuscates the document should be avoided.

We also observed several interesting points from the findings that merit further discussion. First, it seems that all the elements of the ICF content required by the major ethical guidelines and regulations are deemed useful, to a certain degree, to both child participants and their parents. This notice seems to be consistent with the observation in a previous survey of school children’s perspectives, in which some children wanted to know all aspects of the research relevant to their participation [40]. As a matter of fact, different children may have different needs and perspectives; some may want to be informed of all aspects of the trial, while others may prefer to defer decisions to their parents [41, 42]. Second, the parent respondents considered information about payment and/or remuneration as being their last rank of priorities, while they tended to pay more attention to compensation for injury. Our observation suggests that financial issues (except for compensation for injury) might not be a major concern for the parents whose role is to act, as proxy decision-makers, in their child’s best interests and protect their child from assuming unreasonable risks [1]. Third, many respondents suggested that it would have been helpful to them if the investigators had provided a summary schedule of the trial (including how many visits and how much time they need to stay at the clinic/hospital during each visit) and their responsibilities (including what to do and what should/must not do) during trial participation. Such information can be in a form of simplified tables or timeline diagrams given in a one-page short note.

We acknowledge certain limitations of this study so the findings of this study should be interpreted in the context of these limitations. First, it is to be noted that the population in this study was Thai child participants with the diagnosis of hematology or oncology diseases and their parents from a single center in Thailand. Whether their views expressed in this study would be reflective of a broader population is unclear. It is reasonable to assume that some concerns may be dominant in some groups of populations but not in others [43, 44]. Thus, the results of this study may not be representative of those with different socio-economic and cultural backgrounds [45]. Moreover, the extent of information needs may largely be shaped by the level of research risk; individuals participating in minimal-risk research studies may have different needs and perspectives from those involving pediatric drug trials [46–48]. Second, the present study did not take into account the child’s capacity/competency of giving informed assent nor any cognitive or emotional maturity of the child. Rather, the child respondents were enrolled based solely on their age [49]. It is generally accepted that the chronologic age at which children are deemed capable of providing informed assent is about 7 years old onwards. However, there is a possibility that some child respondents whose age was around 7–10 years old might have had the limited capacity necessary to determine the importance of each item in the questionnaire [50–52].

Last but not least, the findings of the present study should not be used to dictate assent/consent requirements for pediatric drug trials. This study was not designed to define the essential elements required for valid informed assent/consent; rather, it aimed to provide some insights into which elements of the ICF content are deemed necessary from the child participants’ and the parents’ perspectives. The results of this study could help investigators and sponsors arrange essential information in order of importance and tailor an IAF/ICF to better suit child participants’ and their parents’ needs, particularly for pediatric drug trials involving children with the diagnosis of hematology or oncology diseases. The items perceived as highly important should be made salient and described comprehensively in an IAF/ICF, while the others with relatively lower importance may be summarized in brief.

## Conclusions

Most of the ICF content requirements were viewed as necessary by both child and parent respondents when making decisions about whether to participate in pediatric drug trials; however, some items were prioritized more highly than others. Risk–benefit information (including direct health benefit, indirect/societal benefit, and post-trial benefit/provision, as well as major foreseeable risk and adverse effects of the drug) was perceived to be of utmost importance among the respondents, so it should be made a prominent component of an IAF/ICF. This empirical data could assist related stakeholders in arranging essential information in order of importance and tailoring an IAF/ICF to better meet the needs of child participants and their parents, particularly for pediatric drug trials involving children with the diagnosis of hematology or oncology diseases.

## Data Availability

The datasets used and/or analyzed during the current study are available from the corresponding author upon reasonable request.

## Abbreviations

CFR: Code of Federal Regulations
FERCAP: Forum for Ethical Review Committees in the Asia and Western Pacific region
IAF: Informed assent form
ICF: Informed consent form
ICH: International Conference on Harmonization
IEC: Independent Ethics Committee
IQR: Interquartile range
IRB: Institutional Review Board
SD: Standard deviation
